# Long-term antihypertensive effects of far-infrared ray irradiated from wooden board in spontaneously hypertensive rats

**DOI:** 10.1186/s12906-016-1040-1

**Published:** 2016-02-08

**Authors:** Chien-Tsong Lin, Ming-Ju Lin, Yung-Pin Chen, Ko-Chao Lee, Kuo-Chin Huang, Shun-Fu Chang, Cheng-Nan Chen

**Affiliations:** 1 Center for General Education, National Formosa University, Yunlin, 632 Taiwan; 2 Department of Wood Based Materials and Design, National Chiayi University, Chiayi, 600 Taiwan; 3 Department of physical Education, Health and Recreation, National Chiayi University, Minsyong, Chiayi County 621 Taiwan; 4 Department of Colorectal Surgery, Department of Surgery, Chang Gung Memorial Hospital; Kaohsiung Medical Cente, Kaohsiung, 833 Taiwan; 5 Department of Orthopaedics, Chang Gung Memorial Hospital Chiayi Branch, Chiayi, 613 Taiwan; 6 Department of Medical Research and Development, Chang Gung Memorial Hospital Chiayi Branch, No. 6, West Sec., Jiapu Rd., Puzi City, Chiayi County 613 Taiwan, ROC; 7 Department of Biochemical Science and Technology, National Chiayi University, Chiayi, 600 Taiwan

**Keywords:** Angiotensin II, Far-infrared ray, Hypertension, Spontaneously hypertensive rats

## Abstract

**Background:**

Far-infrared ray (FIR) has been widely used in promoting health and has been shown to exert beneficial effects in vascular function. The non-thermal effect of FIR has been found to play a significant role in the protective effect on some vascular-related diseases, but its protective effects and use against hypertension have not been clearly presented.

**Methods:**

In the present study, by using a wooden board coated with FIR-irradiated materials, we evaluated the long-term antihypertensive effect on spontaneously hypertensive rats (SHRs) in the environment in contact with the FIR-irradiated wooden board. SHRs were placed on the wooden board with or without FIR radiation for 4 weeks.

**Results:**

The systolic blood pressure (BP) of SHRs undergoing different treatments was measured weekly using a tail-cuff method. FIR radiation significantly reduced the systolic BP of the SHRs along with a decreasing plasma level of angiotensin II and an increasing plasma level of bradykinin. In addition, long-term contact of FIR did not significantly affect the BP in normotensive Wistar Kyoto rats (WKYs).

**Conclusions:**

Our results provided the evidence based on which FIR radiation could be considered an effective non-pharmacological choice for preventing hypertension.

## Background

Hypertension, the persistent increase of blood pressure (BP) above 140/90 mmHg, is a major risk factor for the development of cardiovascular disease, including stroke, myocardial infarction, heart and renal diseases, and cardiac mortality. Although the etiology of hypertension is complex, the renin-angiotensin system (RAS) plays a major role in the pathology of hypertension [1, 2]. Renin catalyzes the conversion of angiotensinogen to angiotensin I, which is the rate-determining step. Angiotensin-converting enzyme (ACE) then catalyzes the conversion of angiotensin I to angiotensin II, leading to the constriction of the arterial vessels and the regulation of systemic BP [3]. Since the increased levels of angiotensin II are closely related to the development and maintenance of hypertension, the inhibition of angiotensin II formation has been widely considered a major strategy for lowering BP in patients with hypertension [4, 5]. Because sustained BP elevation may increase the incidence of atherosclerosis and stroke, it is important to develop alternative strategies for the treatment of hypertension.

The far-infrared ray (FIR) is generally defined as the electromagnetic wave in the wavelength range of 5.6–1000 μm. FIR radiation has been widely applied to a variety of fields as it has positive effects on food preservation [6], health promotion [7], and cardiovascular systems [8, 9]. Some studies indicated that FIR therapy has potentially beneficial effects in the treatment of wound healing [10], diabetes [11], tumor thermal therapy [12], chronic fatigue syndrome [13], and knee osteoarthritis [14]. In addition, several studies also indicated that FIR radiation exerts beneficial effects in the treatment of vascular diseases. FIR radiation improved impaired vascular endothelial function in patients with heart disease [15], decreased endothelial inflammation by induction of heme oxygenase-1 [16], and augmented angiogenesis in the ischemic hind limb [11]. These studies revealed that the potential effects of FIR therapy played a significant role in the protective effect on vascular function.

There is an increasing evidence that the secretion of soluble inflammatory makers play a significant role in hypertension [17, 18]. Soluble intercellular adhesion molecule 1 (sICAM-1), vascular adhesion molecule 1 (sVCAM-1), and sE-selectin are major inflammatory markers and endothelial cell adhesion molecules that regulates the binding and extravasation of leukocytes from the bloodstream to sites of inflammation [19]. The appearance of soluble inflammatory markers in the circulation is thought to be the consequence of their release from the vascular cells because of increased expression [20].

The spontaneous hypertension rats (SHRs) are the most widely used animal model in the study of hypertension-related diseases. The duration, cardiac hypertrophy, renal dysfunction, and cardiovascular complications of SHRs are similar to those observed in human hypertension. In this study, by using the wooden board coated with FIR-emitting materials, we evaluated the long-term effect on SHRs in contact with the FIR-emitting wooden board. Wistar Kyoto rats (WKYs) were used as the control group. These findings provide new insights into the treatment of hypertension using FIR radiation.

## Methods

### The FIR-irradiated wooden board

All of the FIR-irradiated wooden board (53 × 21.5 × 1.5 cm; length × width × thickness) samples used in this study were made by Ua Wood Floors, Inc. (Yunlin, Taiwan). The wooden boards were coated with Nano-paste (ZnO), which was manufactured by Nano-Infinity Nanotech Co., Ltd. (Taoyuan, Taiwan). Using the far-infrared ray testing system (Bruker, VERTEX 70 FT-IR) to test for far-infrared emissivity, the spectrums of the FIR-irradiated wooden board exhibited high emissivity (>0.8) between 4 ~ 14 μm wavelengths (triplicate samples were made for each wooden board, with measurements done by Industrial Technology Research Institute in Taiwan).

### Animal model

The study was performed in accordance with the guidelines for laboratory animals of National Chiayi University. The protocol was approved by the Institutional Animal Care and Use Committee of National Chiayi University. All efforts were made to minimize suffering. Eight-week-old male SHRs and WKYs were obtained from the National Laboratory Animal Breeding and Research Center of the National Science Council, Taiwan. All rats were maintained five per cage at a constant temperature (24 ± 1 °C), with a 12-h dark/light cycle and a standard rat chow diet. After the SHRs had been housed for 10 weeks, their weight ranged from 240–250 g, and systolic BP reached 180 mmHg. The rats were randomly divided into four groups (*n* = 10 per group). Group 1: SHRs on a wooden board without FIR radiation as the control group; group 2: SHRs on a wooden board with FIR radiation; group 3: SHRs on a normal wooden board with atenolol administration as the positive control group; and group 4: SHRs on an FIR-radiated wooden board with atenolol administration. In addition, the WKY rats were divided into group 1 and group 2 for comparing the SHRs in this study.

### Measurement of BP

BP was measured indirectly and non-invasively every week by using the volume-oscillometric tail-cuff method (UR-5000, UEDA, Industries Co., Tokyo, Japan). To get an accurate BP, at least 7 consecutive determinations were recorded. The maximum and minimum values of BP were then eliminated, and the BP was calculated as the average of the remaining values [21].

### Analysis of plasma angiotensin II, bradykinin, and ACE activity

After animal sacrifice, blood samples were collected and kept on ice. The samples were collected and centrifuged at 3000 rpm for 15 min at 4 °C. The plasma was stored at−80 °C until analysis. Both angiotensin II and bradykinin were quantified by respective ELISA kits (Angiotensin II ELISA, Cayman Chemical, Ann Arbor, MI, USA; Bradykinin ELISA, Phoenix Pharmaceuticals, Burlingame, CA, USA) as per the manufacturers’ instructions. ACE activity was determined according to the colormetric method using synthetic sybstrate (FAPGG) [22]. One unit (U) of ACE acitvity is defined as the amount of which released 1 μmol of His-Leu per minute. The specific activity of ACE is expressed as U/L.

### Analysis of plasma inflammatory markers

The levels of sICAM-1, sVCAM-1, and sE-selectin in plasma were determined by using sandwich ELISA (R&D Systems, Minneapolis, MN) according to manufacturer’s protocols [23].

### Statistics analysis

Data were reported as the mean ± standard error of the means (SEM) and evaluated by one-way analysis of variance. Significant differences were established at *P* < 0.05.

## Results

### FIR-irradiated wooden board attenuates BP in SHRs

**Fig. 1:**
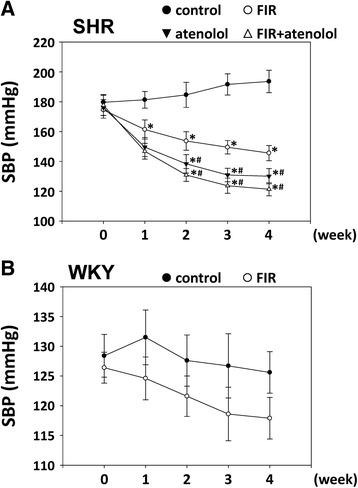
Changes in systolic BP of experimental rats during the 4-week period. **a** The SHRs were randomly divided into four groups (*n* = 10 per group). Group 1: SHRs on a wooden board without FIR radiation as the control group (control); group 2: SHRs on a wooden board with FIR radiation (FIR); group 3: SHRs on a normal wooden board with atenolol administration as the positive control group (atenolol); and group 4: SHRs on an FIR-radiated wooden board with atenolol administration (FIR + atenolol). **b** The WKY rats were randomly divided into two groups (*n* = 10 per group). Group 1: WKYs on a wooden board without FIR radiation as the control group (control); and group 2: WKY rats on a wooden board with FIR radiation (FIR). Data are mean ± SEM. *p < 0.05 vs. control SHRs, #*p* < 0.05 vs. SHRs with FIR irradiation

The SHRs demonstrated already established hypertension at the beginning of the study (week 0). The results from this study, as illustrated in Fig. 1a, demonstrated that at the start of the experiment, the mean values of systolic BP in all the SHRs were similar (174.5 to 179.6 mmHg). The systolic BP in the control SHRs was increased up to 193.6 mmHg at the end of the 4-week period of experiment, whereas SHRs on an FIR-irradiated wooden board started to reduce systolic BP after 1 week, and this effect continued until the 4-week experimental period with gradual reduction in systolic BP. Among the treatment, SHR administration with atenolol reduced the systolic BP more significantly at week 3 and 4 compared with the SHRs treated with FIR only. There were no obvious changes in the systolic BP in WKYs with or without FIR irradiation (Fig. 1b). These experiments suggested that the effect of FIR irradiation on SBP levels has specificity against hypertensive rats.

### FIR irradiation inhibits plasma ACE activity in SHRs

**Fig. 2:**
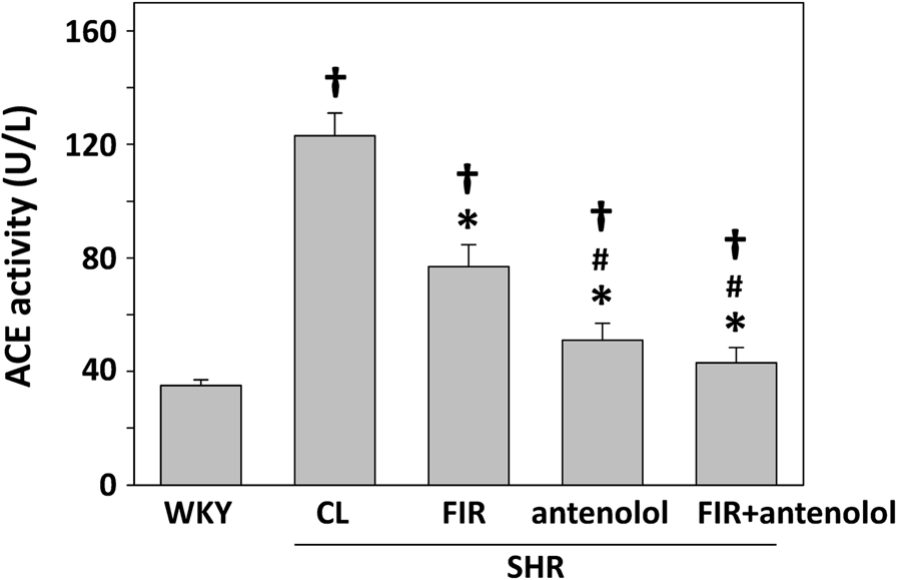
Plasma ACE activity of experimental rats after the 4-week treatments. WKYs: WKYs on a wooden board without FIR radiation; CL: SHRs on a wooden board without FIR radiation; FIR: SHRs on a wooden board with FIR radiation; atenolol: SHRs on a normal wooden board with atenolol administration; FIR + atenolol: SHRs on an FIR-radiated wooden board with atenolol administration. Data are mean ± SEM from *n* = 10 animals per treatment group. *p < 0.05 vs. control SHRs, #*p* < 0.05 vs SHRs with FIR irradiation, †*p* < 0.05 vs. WKY group

Plasma ACE activity in the SHR untreated group was significantly higher than in the WKY group (35.4 ± 3.1 vs. 124.5 ± 8.4 U/L). FIR irradiation decreased ACE activity in the SHR untreated group to 76.7 ± 6.3 U/L in the FIR-irradiated group (Fig. 2). In addition, SHR administration with atenolol further reduced the plasma ACE activity compared with the SHRs treated with FIR only (Fig. 2).

### FIR irradiation appears to inhibit angiotensin II in vivo

**Fig. 3:**
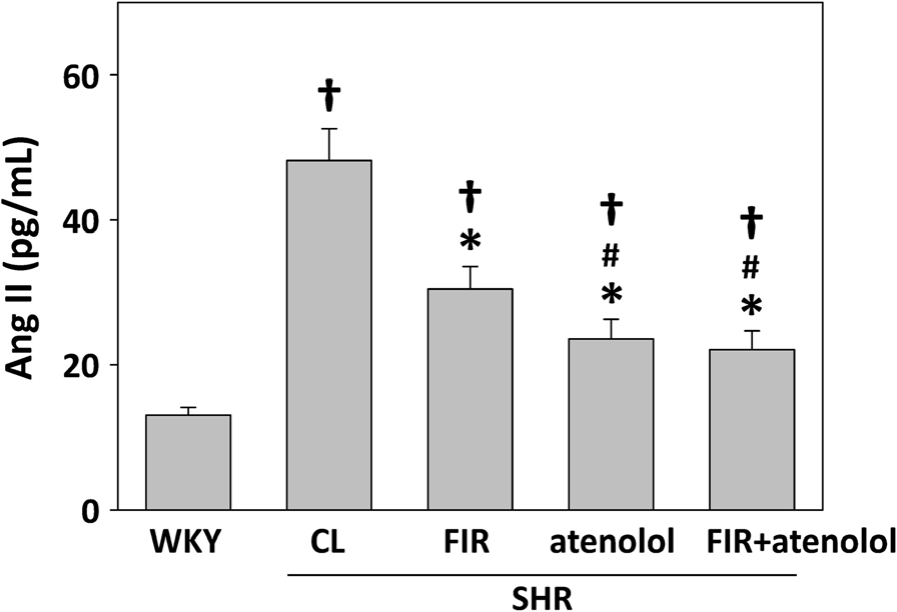
Plasma angiotensin II levels of experimental rats after the 4-week treatments. WKYs: WKYs on a wooden board without FIR radiation; CL: SHRs on a wooden board without FIR radiation; FIR: SHRs on a wooden board with FIR radiation; atenolol: SHRs on a normal wooden board with atenolol administration; FIR + atenolol: SHRs on an FIR-radiated wooden board with atenolol administration. Data are mean ± SEM from *n* = 10 animals per treatment group. *p < 0.05 vs. control SHRs, #*p* < 0.05 vs SHRs with FIR irradiation, †*p* < 0.05 vs. WKY group

**Fig. 4:**
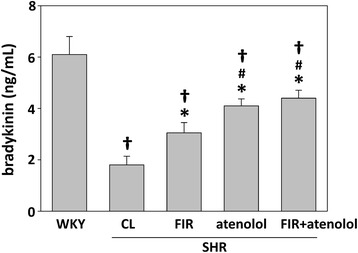
Plasma bradykinin levels of experimental rats after the 4-week treatments. WKYs: WKYs on a wooden board without FIR radiation; CL: SHRs on a wooden board without FIR radiation; FIR: SHRs on a wooden board with FIR radiation; atenolol: SHRs on a normal wooden board with atenolol administration; FIR + atenolol: SHRs on an FIR-radiated wooden board with atenolol administration. Data are mean ± SEM from *n* = 10 animals per treatment group. *p < 0.05 vs. control SHRs, #*p* < 0.05 vs. SHRs with FIR irradiation, †*p* < 0.05 vs. WKY group

The effect of FIR irradiation on the systolic BP of the SHRs was associated with concomitant changes in circulating levels of angiotensin II and bradykinin. FIR irradiation decreased angiotensin II levels from 48.2 ± 5.4 pg/mL in the untreated group to 30.5. ± 3.1 pg/mL in the FIR-irradiated group (Fig. 3). In addition, SHR administration with atenolol further reduced the angiotensin II levels compared with the SHRs treated with FIR only (Fig. 3). The treatment also increased the circulating levels of bradykinin (a molecule metabolized by ACE), which increased from 1.82 ng/mL in the untreated group to 3.05 ng/mL in the FIR irradiated group (Fig. 4). Moreover, the WKY rats had the lowest angiotensin II (Fig. 3) and the highest bradykinin (Fig. 4) levels, compared to all the SHR groups. These results suggest that FIR irradiation may decrease the production of angiotensin II as well as inhibit the degradation of bradykinin, thereby decreasing the systolic BP in SHRs.

### FIR irradiation ameliorates plasma levels of inflammatory markers

**Fig. 5:**
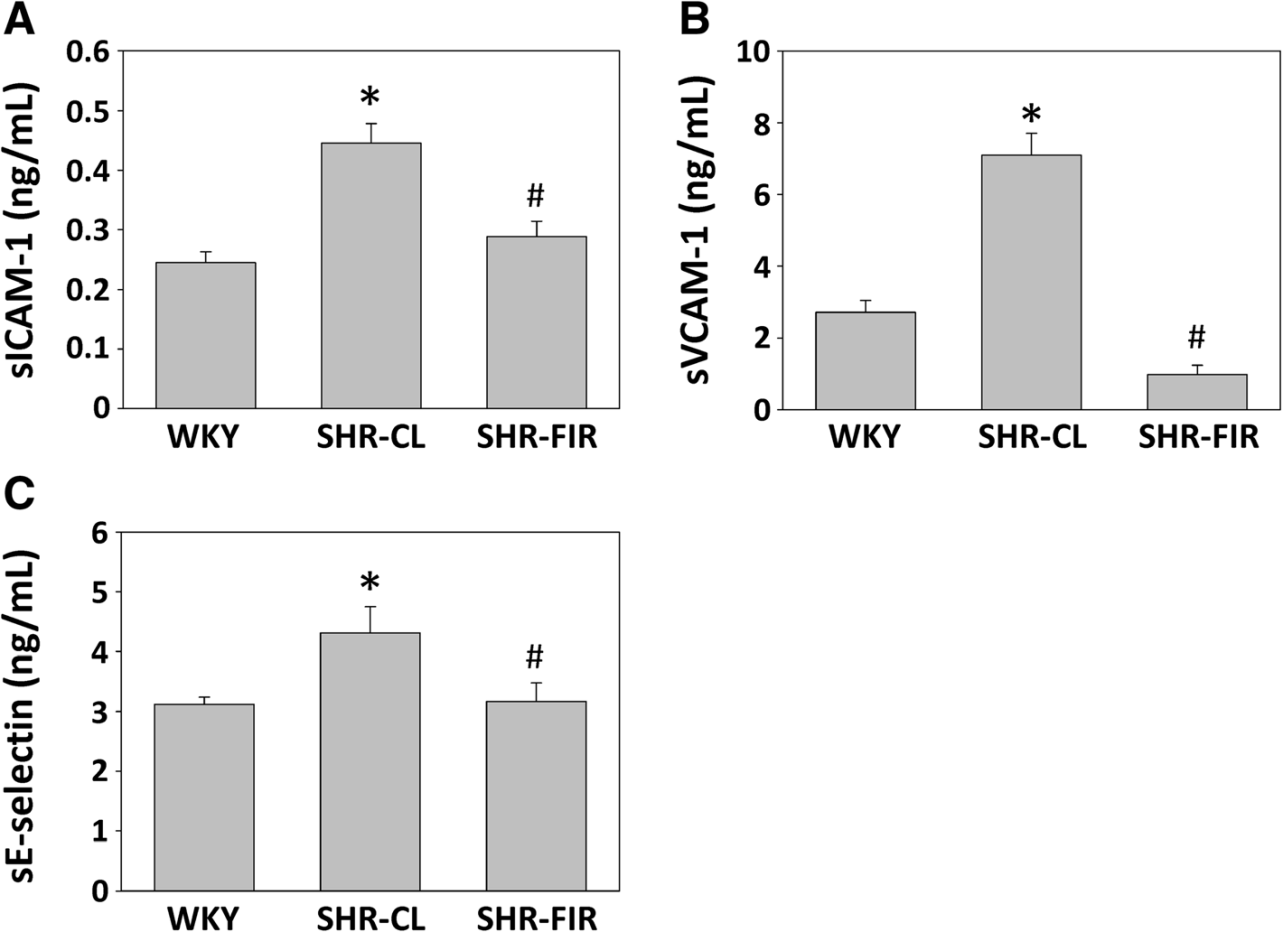
Plasma sICAM-1 (**a**), sVCAM-1 (**b**), and sE-selectin (**c**) levels of experimental rats after the 4-week treatments. WKYs: WKYs on a wooden board without FIR radiation; SHR-CL: SHRs on a wooden board without FIR radiation; SHR-FIR: SHRs on a wooden board with FIR radiation. Data are mean ± SEM from *n* = 10 animals per treatment group. *p < 0.05 vs. WKY group, #*p* < 0.05 vs. SHRs with FIR irradiation

We further evaluated the effect of FIR irradiation on plasma levels of sICAM-1, sVCAM-1, and sE-selectin in SHRs. SHR untreated group exhibited an increase in sICAM-1, sVCAM-1, and sE-selectin levels compared to WKY rats (Fig. 5). However, SHRs treated with FIR showed decreased sICAM-1, sVCAM-1, and sE-selectin levels compared to untreated group (Fig. 5).

## Discussion

Recent findings showed that FIR radiation might play a role in the long-term protective effect on vascular function by reducing several cardiovascular risk factors such as high BP. Our studies with FIR irradiation in SHRs demonstrated for the first time that rats placed on FIR irradiated wooden board indeed showed a systolic BP reducing effect in the hypertensive rats in a time-dependent manner. The FIR also decreased angiotensin II and increased bradykinin production in the plasma of the treated SHRs, suggesting the possible mechanisms for the systolic BP reducing effect of FIR. Thus, FIR could prove to be a novel method for management of hypertension.

SHRs are the animal model most frequently used to evaluate the in vivo antihypertensive effect of different treatments such as drugs or dietary components. Various studies performed to determine the antihypertensive effects of food-derived bioactive peptides have used SHR animals as a model system [24, 25]. To verify the validity of the experimental protocol, both a negative and a positive control group were included. The underlying pathological mechanisms of the hypertensive effect in SHRs include increased activity of the renin-angiotensin system as well as increases in vascular inflammation and oxidative stress [26, 27]. Hence, SHRs are a suitable model to study the in vivo antihypertensive efficacy of FIR irradiation. In the present study, systolic BP increased in the control SHR group during the 4-week period of the study. In addition, the systolic BP was significantly decreased in the positive control group (which received atenolol administration) when compared to the negative control group. Thus, the negative and positive control groups confirmed the validity of the animal model chosen in this study.

The increase in BP is directly due to the vasoconstrictive actions of angiotensin II produced from angiotensin I in the presence of ACE [28, 29]. Angiotensin II significantly increased the BP by the activation of angiotensin II receptors in the vascular smooth muscle cells, resulting in increased vasoconstriction, decreased renal blood flow and renal tubular sodium re-uptake, and increased vasopressin secretion [30–32]. In our present study, FIR radiation appears to act through angiotensin II, leading to lowered BP in SHRs. FIR radiation reduced the plasma and angiotensin II levels with a corresponding increase in bradykinin. The plasma levels of ACE activity tended to be positively related to BP changes, which is consistence with the earlier work that have shown that ACE levels could be used as a hypertension marker in SHRs [33]. Our result is further confirmed by the fact that FIR-irradiated SHRs had significantly reduction of plasma ACE activity when compared to the untreated control, suggesting a role of elevated plasma ACE activity in modulating hypertension conditions. Thus, the antihypertensive effects of FIR radiation may be mediated by downregulation of ACE with a consequent reduction in the plasma levels of the vasoconstrictor angiotensin II.

It has been showed that FIR therapy has been applied to various clinical fields, particularly including vessel-related disorders [34]. FIR radiation enhanced blood flow retrieval and blood vessel formation in ischemic hind limbs by enhancing the homing of endothelial progenitor cells and lowering the oxidative stress in ischemic tissue. Direct FIR radiation could also ameliorate high-glucose-induced oxidative stress, suppress cellular senescence, and improve endothelial progenitor cell functions [11]. Although the mechanism by which FIR radiation has a potential for the treatment of vascular diseases is still unclear, the improvement of endothelial cell function is currently considered as a mechanism [35]. In addition, increasing evidence suggests FIR therapy could exert beneficial effects in the cardiovascular system through the NO-related pathway [36]. FIR therapy could upregulate eNOS expression and increase angiogenesis in an apolipoprotein E-knockout mouse model [37]. These findings suggest that FIR may provide some novel rationales for its potential clinical impact on vascular protection.

Vascular dysfunction or damage can be evaluated by the measurement of inflammatory markers released from vascular cells, such as sICAM-1, sVCAM-1, and sE-selectin [38]. Several studies have demonstrated that the plasma levels of inflammatory markers are increased in patients with hypertension, type 2 DM, and atherosclerosis, all of which are well-established risk factors for cardiovascular diseases [39–41]. Higher levels of soluble inflammatory markers are associated with increased cardiovascular risk in hypertension, presumably because such high levels reflect chronic, low-grade vascular inflammation [18]. FIR therapy has been reported to exert a potent anti-inflammatory effect via induction of HO-1 release by human umbilical vein endothelial cells [16]. Down-regulation in these markers may confer a decrease in cardiovascular risk, although prospective data are needed to test this hypothesis. In the current study, FIR irradiation decreased levels of the inflammatory markers sICAM-1, sVCAM-1, and sE-selectin, suggesting a specific anti-inflammatory property of FIR in vivo. Since SHR untreated group has increased levels of plasma inflammatory markers which may contribute to endothelial dysfunction, it is suggested that controlling the inflammatory responses with FIR irradiation could potentially improve the vascular function and reduce inflammation.

## Conclusions

This study provides evidence of the antihypertensive effect of FIR irradiation in SHRs. We found that the treatment of SHRs with FIR irradiation induced a decrease in BP. Since FIR substantially decreased the SHR plasma ACE activity and angiotensin II level, and increased the bradykinin level, we believe that the mechanism of the antihypertensive effect of FIR on SHRs may be related to the ACE inhibitory effect. Our results suggest a possible beneficial effect of FIR on cardiovascular function and thus provide a mechanistic basis for the use of FIR as an antihypertensive remedy in folk medicine.

## Abbreviations

BP: blood pressure
RAS: renin-angiotensin system
ACE: angiotensin-converting enzyme
FIR: far-infrared ray
SHR: spontaneous hypertension rat
WKY: Wistar Kyoto rat
